# The Effect of UV Exposure on Selected Surface Parameters of Wood Containing Graphene Oxide

**DOI:** 10.3390/molecules30244730

**Published:** 2025-12-10

**Authors:** Izabela Betlej, Aneta Bombalska, Karolina Lipska, Miron Kaliszewski, Piotr Borysiuk

**Affiliations:** 1 Institute of Wood Sciences and Furniture, Warsaw University of Life Sciences—SGGW, 159 Nowoursynowska St., 02-776 Warsaw, Poland; karolina_lipska@sggw.edu.pl; 2 Institute of Optoelectronics, Military University of Technology, Gen. S. Kaliskiego 2 St., 00-908 Warsaw, Poland; aneta.bombalska@wat.edu.pl (A.B.); miron.kaliszewski@wat.edu.pl (M.K.)

**Keywords:** pine and birch wood, graphene oxide, UV aging, FTIR, contact angle, color change

## Abstract

The study aimed to investigate the effect of UV radiation on the chemical and physical changes in pine and birch wood surfaces impregnated with graphene oxide. The samples were exposed to UV radiation with an intensity of 550 W/m^2^ for 16, 32, and 48 h. FTIR analysis revealed photodegradation of lignin in both wood species. It was indicated that graphene oxide impregnation may slow down the rate of lignin oxidation. Graphene oxide impregnation also affected the change in the surface contact angle, with the differences being more pronounced in birch wood than in pine. Color measurements showed that graphene oxide impregnation significantly altered the initial color of the wood (darkening and a shift towards green and blue), and UV radiation intensified the color changes, especially in birch wood.

## 1. Introduction

Wood is an environmentally friendly material for construction and finishing. It is valued in architectural applications for its aesthetic appeal and functional properties. Despite its many advantages, wood, like any other material, ages, and its physical and mechanical properties change over time. Sunlight is one of the most important natural factors to which wood is exposed. Sunlight, or more precisely UV radiation, which is its electromagnetic component, is responsible for structural and chemical changes in the surface of wood, often manifested by a change in surface color, loss of gloss, or weakening of the structure [1,2]. UVA and UVB radiation are considered the most important from the perspective of materials science. They cause lignin oxidation, hemicellulose degradation, weakening of cellulose bonds, and surface cracking resulting from mechanical stress [3]. The degradation of cell wall components makes wood more absorbent and susceptible to moisture. Many factors influence the rate of wood photodegradation. The basic ones include the type of wood and the intensity of radiation and exposure to additional atmospheric conditions such as snow, rain, or pollution in the form of nitrogen and sulfur oxides [4,5].

The basic process of protecting wood against progressive photodegradation is chemical impregnation with agents that protect against the harmful effects of UV radiation. Pigments that reflect UV radiation, impregnated with antioxidant additives and lignin stabilizers, are used for this purpose [6]. Using physical protection in the form of covers also slows down the aging process of wood. In places where wood is most exposed to UV radiation, thermally modified wood, which is more resistant to aging processes, can be used as an alternative [7]. The use of species that are naturally resistant to UV radiation may also be a good solution.

The chemical agents currently available for protecting wood against photo-oxidation are mainly based on benzotriazole [8] and titanium dioxide [9], which act as a physical barrier, protecting wood cells from lignin damage. The literature also contains data on the use of natural polyphenolic compounds (tannins), which have antioxidant properties that prevent the oxidation of wood components [10]. An interesting solution for protecting wood against the destructive effects of UV radiation may be nanomaterials, such as graphene oxide (GO) [11]. Graphene oxide contains numerous functional groups that give it high chemical reactivity and the ability to combine with other substances [12]. The work of numerous authors has shown that GO can scatter and absorb UV radiation, resulting from the high concentration of conjugated π bonds [13]. The optical properties of GO are well documented, making it a promising material in the context of light protection [14]. This nanomaterial is highly reactive due to its numerous hydroxyl, epoxy, and carboxyl groups, enabling it to form stable bonds with organic and inorganic compounds. Literature data indicate that wood impregnated with graphene oxide is characterized by a reduction in the number of microcracks and surface degradation by up to 40–60%, as well as better preservation of strength parameters, especially hardness and stiffness [15]. Research conducted by Aqlil et al. [16] has shown that the graphene oxide layer in impregnated wood acts as a screen protecting lignin and hemicellulose from photodegradation.

Contemporary forestry is increasingly looking to innovative materials that can impart new properties to wood. One of the most promising directions is the use of nanomaterials, in particular graphene oxide. This remarkable material allows for easy chemical modification and integration with traditional raw materials. Numerous authors are researching the use of graphene or graphene oxide in wooden materials [17,18,19]. Tasi et al. [20] showed that functionalized graphene oxide nanoplatelets on the surface of wood increase its fire resistance. Gul et al., on the other hand, found that the addition of GO to resin in MDF production improves the mechanical and physical properties of these MDF boards. In their research, Zhang et al. [21] showed that GO in cellulose coatings can block a high percentage of UV radiation, thus providing cellulose materials with better protection against radiation.

This article aimed to analyze the effect of UV radiation on the surface properties of pine and birch wood impregnated with graphene oxide, with particular emphasis on changes in the structure of polymers, which are the building blocks of wood cell walls. The study aimed to investigate whether the duration of UV exposure on the wood surface affects changes in the deformation of chemical bonds in polymers. Additionally, it was determined whether UV radiation affects the differences in wettability of GO-impregnated wood and changes in the surface color. The use of graphene oxide as a functional additive to enhance the durability of wood is a crucial issue that warrants further development. Atmospheric factors, such as solar radiation, promote rapid photoaging of the wood surface, altering its physical properties, including wettability and hygroscopicity. A change in wettability, in turn, can lead to dangerous biodegradation caused by microorganisms. The search for new methods and ways to limit the rate of photodegradation is important for maintaining the durability of wood during use. The use of graphene oxide as a wood preservative is an innovative approach to UV protection. Nanomaterials, such as graphene oxide and metal oxide nanoparticles, are a new generation of protective agents that, thanks to their nanostructure, form an effective barrier against UV radiation and extend the lifespan of materials. The use of this type of material in impregnating pine and birch wood represents an innovative approach that distinguishes it from traditional protection methods. The knowledge gained on whether graphene oxide can act as a protective component in wood, increasing its resistance to weather conditions and UV radiation, seems important. Positive results could open up prospects for using GO in the production of wood or wood-based materials with increased service life.

It should also be noted that, unlike previous studies, this study provides detailed experimental data on the photodegradation of two wood species that differ in anatomical structure and physical properties, allowing for a broader perspective on the potential effectiveness of protection. At the same time, pine and birch are commonly used species in the wood industry, which gives the research practical and applicative significance.

## 2. Results and Discussion

### 2.1. Assessment of the Effect of UV Radiation on Changes in the Structure of Cell Wall Polymers

**Figure 1:**
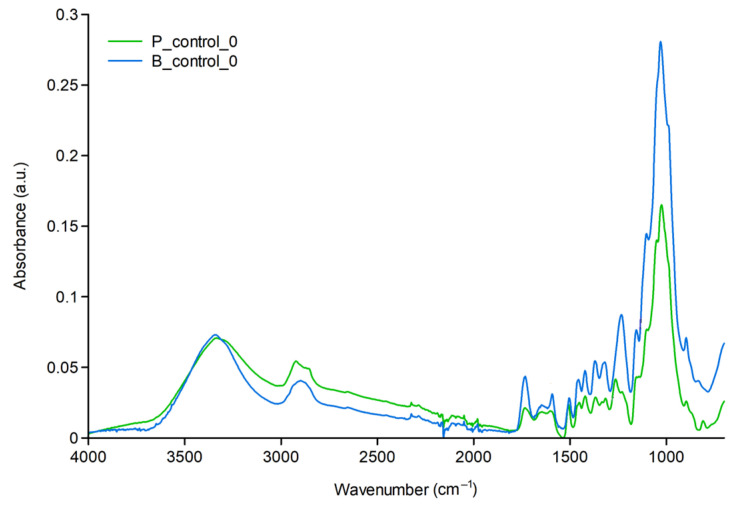
FTIR spectra of unmodified pine and birch samples.

The infrared spectra of unmodified wood samples are shown in Figure 1. In the fingerprint region, they exhibit characteristic peaks that differ in intensity and position between the tested birch and pine wood species.

Wood degradation is caused by the formation of free radicals during exposure to environmental conditions, including UV radiation. All components of wood are susceptible to degradation under the influence of these factors. The energy of UV radiation is sufficient to break chemical bonds in the wood structure. Chemical analysis of aged wood, influenced by various factors, reveals degradation of lignin and hemicellulose, as well as depolymerization of cellulose. The consequence of photochemical reactions is the loss of methoxyl groups (−OCH_3_) in lignin, the dissociation of C−C bonds, and the formation of carbonyl groups (−C=O). The latter are the cause (resulting from the formation of chromophores) of the change in the color of wood under the influence of sunlight. The most significant contribution to the FTIR spectrum is made by chromophore groups in the ranges: 3300–4000 cm^−1^, attributed to −OH group vibrations; 2800–3000 cm^−1^, attributed to −CH stretching vibrations; 1700–1800 cm^−1^, C=O stretching vibrations; 900–1100 cm^−1^, C−O. Bands with a smaller share in the spectrum, but significant in the context of wood photodegradation, are: 1505 cm^−1^ (C=C of the aromatic skeleton), 1462, 1422 cm^−1^ (deformation −CH) [22,23].

**Figure 2:**
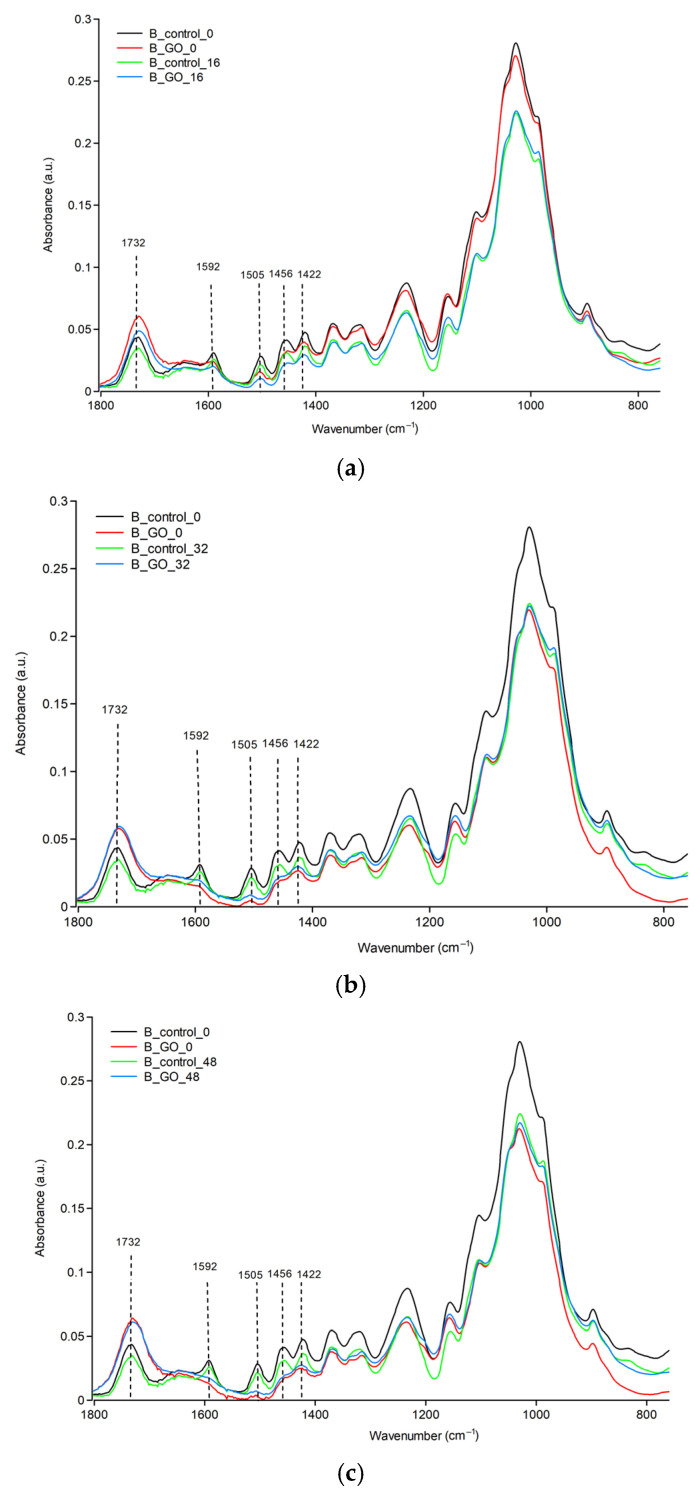
FTIR spectrum of birch samples: (**a**) irradiated for 16 h; (**b**) irradiated for 32 h; (**c**) irradiated for 48 h.

Figure 2a–c show FTIR spectra of birch samples, pure and coated with GO, exposed to UV irradiation for 0, 16, 32, and 48 h. Previous reports have shown that the lignin component in wood is sensitive to photodegradation, and peaks corresponding to the functional groups in the spectral region of 1800 to 700 cm^−1^ exhibit noticeable changes [24]. As seen in Figure 2, the lignin absorption peak at 1505 cm^−1^, which corresponds to C=C stretching aromatic ring in lignin [22,25], decreased gradually with increasing UV irradiation. The peak was almost invisible after 48 h of UV irradiation (Figure 2c). Moreover, peaks at 1592 cm^−1^ (C=C stretching skeletal vibrations of the aromatic ring in lignin) and 1456 cm^−1^ (asymmetric C-H deformations in lignin) [24,26] showed the same decreasing trend. This band (1505 cm^−1^) is considered one of the indicators for monitoring lignin degradation. The degradation of lignin was accompanied by the successive increase in absorption at 1732 cm^−1^, which is assigned to the stretching vibrations of the carbonyl group as a result of wood (lignin) oxidation. An increase in this peak is observed for all samples, depending on the UV radiation dose. Samples coated with GO also exhibit an increase in this peak, indicating the effect of UV radiation on the wood, despite its presence.

**Figure 3:**
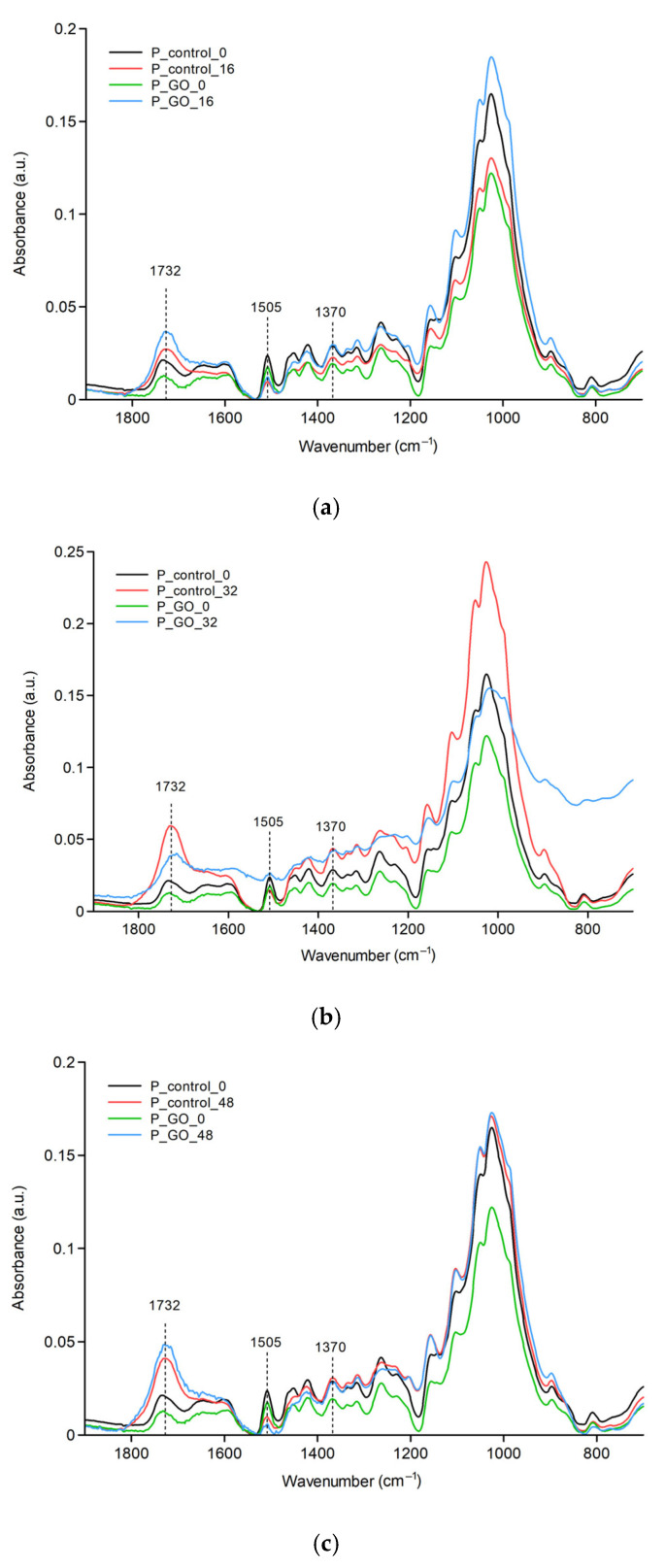
FTIR spectrum of pine samples: (**a**) irradiated for 16 h; (**b**) irradiated for 32 h; (**c**) irradiated for 48 h.

Figure 3a–c show FTIR spectra of pine samples, pure and coated with GO, exposed to UV irradiation for 0, 16, 32, and 48 h. The effect of UV on pine samples is similar to that on birch samples. An increase in the C=O band and a decrease in C=C are observed. The most significant changes also occur after 48 h of irradiation.

To determine the degree of wood photodegradation and the impact of GO on this process, coefficients are calculated that specify the degree of changes occurring in the wood structure under the influence of aging factors. These coefficients determine the degree of increase in the C=O carbonyl group (R1) and the decrease in C=C bonds (R2). The coefficients were calculated by comparing the absorbance intensities of the carbonyl peaks, C=O (1732 cm^−1^) and C=C (1505 cm^−1^), to the constant peaks, C−H (1370 cm^−1^) and C−O−C (1157 cm^−1^), as internal standards: R1 = A_1732_/A_1370_ and R2 = A_1505_/A_1370_ for samples containing GO and without GO [22,27]. The method of determining peak heights was carried out according to [27]. Peak heights were measured using OMNIC 9.0 software (Thermo Fisher Scientific, Waltham, MA, USA). Baseline lines connecting the lowest points on both sides of the peak in question were plotted for each peak. The R1 and R2 coefficients determine the relative ratio between the C=O and C=C peaks and the internal standard C−H peak. A variant of the coefficients related to the C−O−C peak (1157 cm^−1^) was also calculated. The results are presented in Table 1.

**Figure 4:**
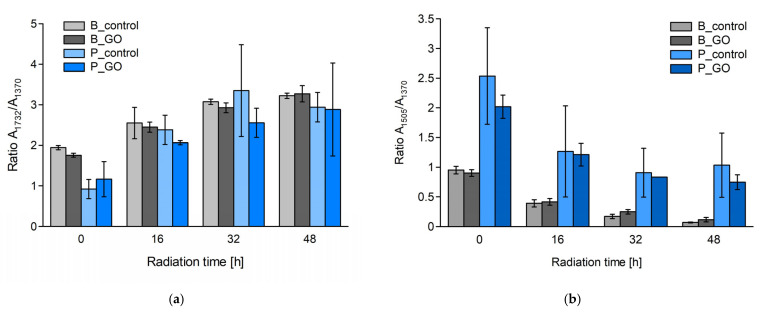
(**a**) C=O band formation (A_1732_/A_1370_) as a function of irradiation time (R1); (**b**) C=C band decay (A_1505_/A_1370_) as a function of irradiation time (R2). B—birch, P—pine.

The degree of change in the veneer structure is illustrated in Figure 4a,b. They show the R1 and R2 coefficients for both sample variants. In both cases, a trend related to wood photodegradation is observed, specifically an increase in the C=O peak and a decrease in the C=C peak. The potential impact of GO on the degree of lignin oxidation would be visible for the −OH, C=O, and C=C bonds. According to Gallegos-Perez et al. [28], irradiation with different doses causes a reduction in GO, which is observed as the disappearance of the −OH and C=O bands, while an increase in C=C and −C−H is recorded. As can be seen, these are opposite effects to those observed in wood photodegradation. If we consider the C=O peak, the overlapping effects of GO and lignin irradiation should theoretically cause a slower increase in the C=O peak for GO samples. However, it is not known which reaction is favored (occurs faster, easier, and with greater efficiency).

**Figure 5:**
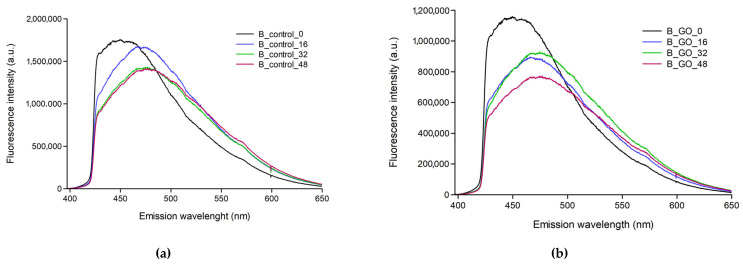

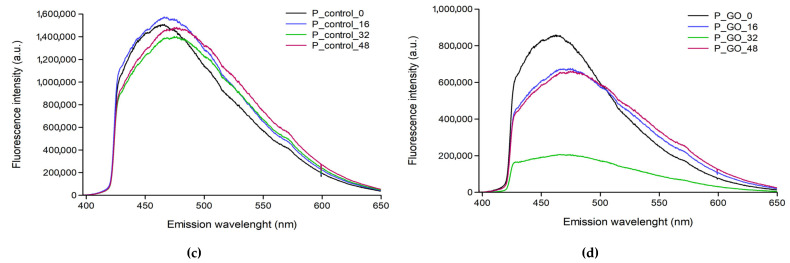
Emission spectra of birch samples as a function of exposure time: (**a**) birch—control; (**b**) birch—impregnated with GO; (**c**) pine—control pine; (**d**) pine—impregnated with GO.

The fluorescence characteristics of the tested samples were also determined (Figure 5a–d). Emission spectra were recorded for excitation at 375 nm. For irradiated birch samples (Figure 5a) without graphene and covered with GO (Figure 5b), a shift in the emission band towards longer wavelengths is observed after irradiation. The maximum emission of pure birch was recorded at a wavelength of 450 nm. With increasing irradiation dose, a shift in the emission peak to 479 nm was observed. After 48 h of irradiation, the peak intensity decreased by 20%. For GO-coated samples, this decrease was 40%. In the case of pine samples (Figure 5c,d), no trend as straightforward as that for birch was observed. Irradiation alone causes the emission maximum to shift from 466 nm to 475 nm only after 32 h of irradiation (Figure 5c). No dose-dependent decrease in fluorescence intensity is observed for pine samples. For samples coated with GO (Figure 5d), a 23% decrease in fluorescence emission intensity is observed after 48 h of irradiation. The shift in the emission peak can be explained by the formation of other fluorophores (as a result of the breakdown of chemical bonds during irradiation), which absorb different wavelengths than unchanged lignin, cellulose, and hemicellulose. However, the results obtained so far require further work using pure lignin and cellulose samples in order to determine whether the observed spectral shifts and intensity changes are related to the degradation of wood components.

### 2.2. Assessment of the Effect of UV on Changes in the Wettability of Birch and Pine Wood Impregnated with GO

**Figure 6:**
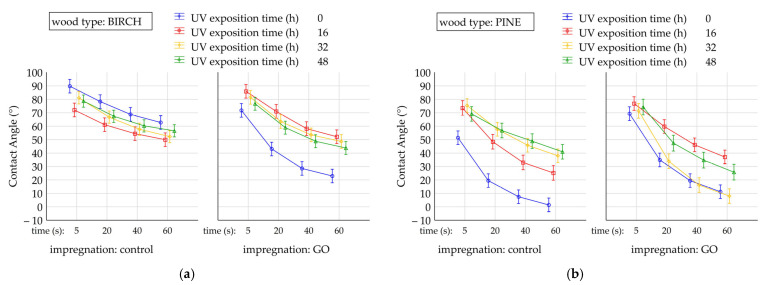
Estimated marginal means (±95% CI) of contact angle after 5, 20, 40 and 60 s from drop deposition, for birch (**a**) and pine (**b**) wood, in native and graphene oxide (GO) impregnated variants, after different durations of UV exposure (0 h, 16 h, 32 h, 48 h), obtained from the GLM analysis.

Figure 6a,b shows the change in the wetting angle over time for native birch (Figure 6a) and pine (Figure 6b) wood, as well as GO-impregnated wood, under different exposures to UV radiation (0 h, 16 h, 32 h, 48 h). The decrease in the wetting angle over time after applying a drop to the surface is a natural phenomenon in wood, related, among other things, to water absorption processes [29]. However, a comparison of these changes for variants differentiated by variable factors (exposure time, impregnation, wood species) allows the significance of these factors to be identified. The wettability of birch and pine decreases over time, but changes in the wettability of both native and GO-impregnated wood surfaces are more noticeable for pine than for birch. The reason for this phenomenon can be found in the difference in the anatomical structure of the two wood species. Changes in the wetting angle may also depend on the chemical composition of the surface, in particular on the presence of natural extractives and chemical photodegradation of the surface due to UV radiation absorption [30].

Changes in the wettability of GO-impregnated birch wood that has not been exposed to UV radiation, observed over a period of up to 60 s, show significant differences compared to non-impregnated wood. A more dynamic decrease in the wettability of wood not exposed to UV radiation may be attributed to the presence of numerous functional groups on the surface of graphene oxide, which bind water more readily and render the wood surface more hydrophilic [31]. Exposure of GO-impregnated wood to UV radiation reveals significant differences compared to native wood, which also undergoes the aging process. However, no significant effect of exposure time on differences in surface wettability was observed. In the case of pine samples, a similar trend in the change in wettability over time, up to 60 s, was observed for both impregnated and native wood. However, while statistically significant differences in wettability were observed between weathered and non-weathered control wood, the differences in wettability were not as pronounced in GO-impregnated wood. Changes in the wettability of GO-impregnated birch wood that has not been exposed to UV radiation, observed over a period of up to 60 s, show significant differences compared to non-impregnated wood. A more dynamic decrease in the wettability of wood not exposed to UV radiation may be attributed to the presence of numerous functional groups on the surface of graphene oxide, which bind water more readily and render the wood surface more hydrophilic [31]. Exposure of GO-impregnated wood to UV radiation reveals significant differences compared to native wood, which is also subjected to the aging process. However, no significant effect of exposure time on differences in surface wettability was observed. In the case of pine samples, a similar trend in the change in wettability over time, up to 60 s, was observed for both impregnated and native wood. However, while statistically significant differences in wettability were observed between weathered and non-weathered control wood, the differences in wettability were not as pronounced in GO-impregnated wood. The wettability of pine wood containing graphene oxide is initially similar to that of native wood. At the same time, it can be observed that the exposure time of pine wood containing GO may affect changes in the wettability of its surface. The effect of graphene oxide on the wettability of wood varies depending on the wood species, and the degree of this change depends on many factors, including the anatomical structure of the wood, the concentration of graphene oxide, and its degree of adhesion to the cell walls or its binding with the cell wall polymers [32,33].

Table 2 presents an analysis of the percentage influence of individual variables (graphene impregnation, type of wood, UV exposure time, and time after placing a droplet) and their interactions on surface wetting angles. It should be noted that all factors and most interactions between them (except for A × B × D, A × C × D, and B × C × D) showed a statistically significant effect (*p* < 0.05). Overall, it can be concluded that among the analyzed variables, the time after placing a droplet (X = 44.38%) and the type of wood (X = 18.34%) had the most significant impact on the wetting angle. These were the dominant factors. UV exposure time (X = 6.36%) and the interaction between graphene impregnation, type of wood, and UV exposure time (X = 7.63%) also had a relatively significant impact. For comparison, the impact of graphene impregnation alone was only 1.52%, indicating that it has a minor effect on the wetting angle of the sample surfaces. It is also worth noting that the percentage of factors not included in the study was significantly higher, at 9.62% error.

### 2.3. Assessment of the Impact of UV on Changes in Wood Color

**Figure 7:**
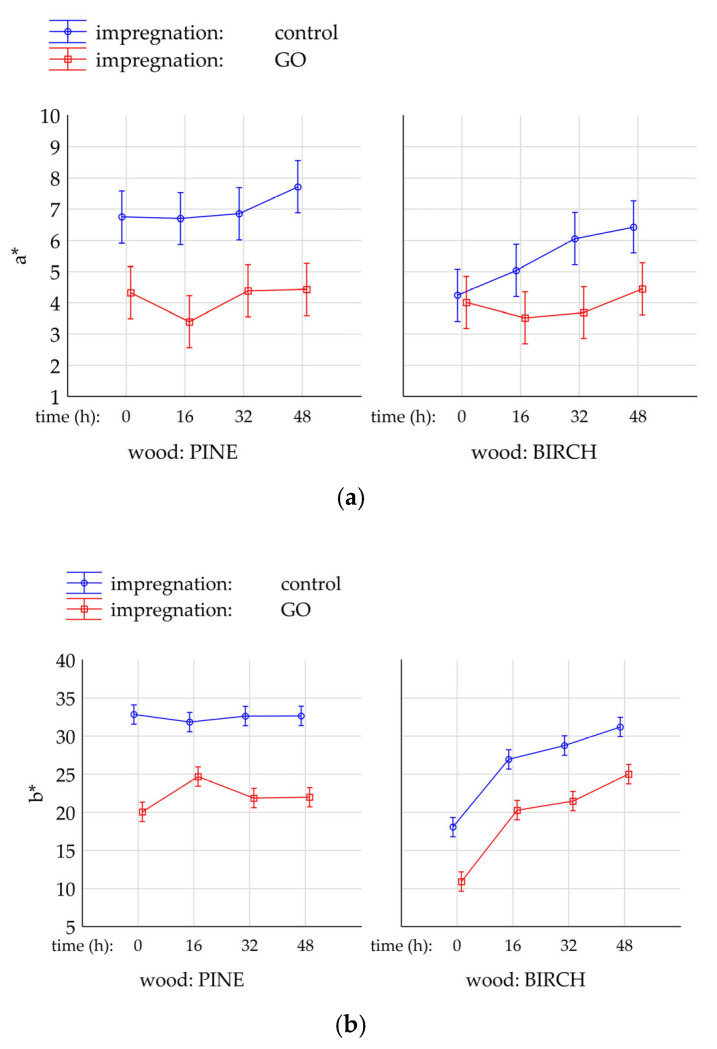

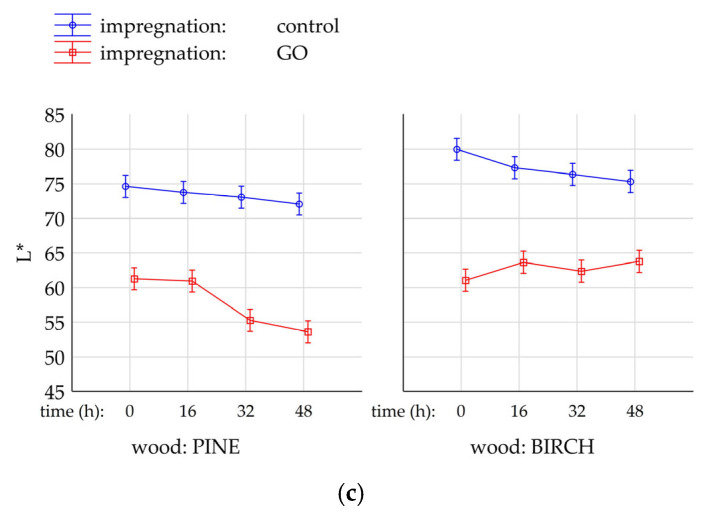
Estimated marginal means (±95% CI) of color parameters for birch and pine wood, in native and graphene oxide (GO)-impregnated variants, after different durations of UV exposure (0 h, 16 h, 32 h, 48 h). (**a**)—parameter a*, (**b**)—parameter b*, (**c**)—parameter L*.

**Figure 8:**
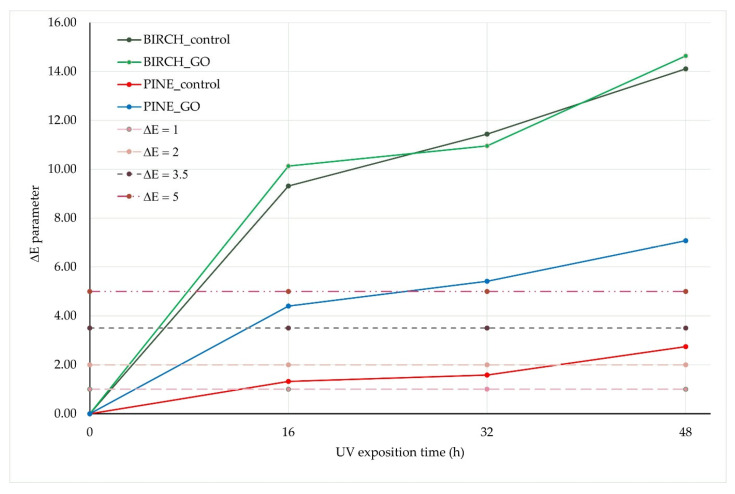
Value of total color difference (∆E) for birch and pine wood, in native and graphene oxide (GO)-impregnated variants, after different durations of UV exposure (0 h, 16 h, 32 h, 48 h).

Figure 7a–c shows the results of color changes on the surface of birch and pine wood samples (native and impregnated with graphene oxide) under the influence of UV radiation. The color change was assessed after exposure times of 0, 16, 32, and 48 h. As can be easily seen, the wood impregnation process itself affects the color and brightness of the surface. Pine and birch samples not exposed to UV rays show a statistically significant difference in the color parameters a*, b*, and L* between the native and GO-impregnated variants. Only in the case of the parameters of the birch wood surface were no statistically significant differences observed between the native and graphene oxide-impregnated samples (not subjected to photoaging). In the remaining cases, GO impregnation caused a decrease in the values of the L, a, and b parameters, which means that in the case of the L parameter, the samples have a darker color, in the case of the a parameter, there is a shift towards green (red-green axis), and for the b parameter, a shift towards blue (yellow-blue axis). The impact of UV radiation causes a progressive change in the color of the control wood surface. The longer the exposure time, the more visible the color difference (ΔE) becomes (Figure 8).

For non-impregnated variants, after 48 h of UV radiation exposure on the birch wood surface, the color change is visible to the observer (5 < ΔE). In the case of pine wood, ∆E after 48 h of UV exposure was less than 3.5, suggesting that a standard observer may not notice the color difference. The color change (∆E) of the surface of pine and GO-impregnated birch after 48 h of exposure to UV radiation was 7.3 and 14.8, respectively (Figure 8). For non-impregnated variants, after 48 h of UV light exposure on the surface of birch wood, the color change is visible to the observer (5 < ΔE). In the case of pine wood, ∆E after 48 h of UV exposure was less than 3.5, suggesting that a standard observer may not notice the color difference. As can be seen, UV exposure to the wood surface intensifies the color change in impregnated wood. The reason for this phenomenon can be found in the changes occurring in the GO structure. According to Ji et al. [34], UV radiation can oxidize and damage the GO structure to a certain extent. The change in the color of impregnated wood visible to the observer obtained in this study may not necessarily be associated with changes leading to damage to the structure of wood polymers, as confirmed by spectrometric studies. According to research conducted by Łukawski et al. [32], GO contained in wood cells significantly improves the resistance of wood to UV radiation. However, several factors enhance the resistance of wood containing GO to photoaging, including the method of graphene oxide deposition in wood [35] and the size and type of functional groups on its surface [36].

Native wood degrades under the influence of UV radiation, changing color, losing its shine, and turning yellow or gray. Additionally, lignin and hemicellulose decompose. Graphene oxide (GO), on the other hand, acts as a protective barrier, absorbing and scattering some of the UV radiation, thereby limiting the degradation of the material; however, GO itself may undergo specific chemical changes. Studies conducted by Wan et al. [37] showed that wood coated with graphene oxide after 600 h of aging tests, including exposure to fluorescent UV radiation, showed significantly less change in surface color and chemical composition than the original wood samples.

The presented studies show that UV radiation causes color changes in both native wood and wood impregnated with graphene oxide. At the same time, it was noted that the color change in control birch wood was more dynamic than in pine wood, which may be related to the anatomical structure and chemical composition of pine wood and the presence of non-structural components (resins) that are not found in birch wood.

When comparing the control and impregnated birch samples, it is clear that the differences in surface color change are visible to the observer. In contrast, the case of the control and impregnated pine is slightly different. The control pine samples exposed to UV change color, but after 48 h of UV exposure, the change in the color of the pine surface is not significant and may not be noticeable to the untrained eye. The addition of graphene oxide mainly causes a change in the color of the early wood zone. Exposure of the wood-GO surface to UV radiation probably also causes changes in the structure of the graphene oxide itself, as indicated by the fact that the color change of the surface of impregnated pine wood after UV exposure is more visible to the observer than in the case where GO is not present in the wood.

Table 3 presents an analysis of the percentage influence of individual variables (IMP, wood type, exposure time) and their interaction on the color system coordinates (L, a, b). It is worth noting that all analyzed factors demonstrated a statistically significant influence (*p* < 0.05). In general, it can be concluded that among the analyzed variables, graphene impregnation had the most significant percentage impact on the color system coordinates (X = 72.46% for L; X = 23.02% for a; X = 40.24% for b). In each case, it was the dominant factor. In turn, the interactions between these factors were varied.

In most cases (except for A × B, A × C for “L” and A × C, B × C, A × B × C for “a”), they also had a statistically significant impact (*p* < 0.05) on the values of the color system coordinates. However, this impact was significantly smaller than that of the dominant factor. The highest percentage impact of graphene impregnation on the “L” coordinate resulted from the fact that it concerns a change in color brightness, while the smallest percentage impact related to the change in color from red to green (coordinate “a”). Regarding the “b” coordinate (change in color from yellow to blue), the type of wood (X = 11.01%) and exposure time (X = 16.45%) had the highest percentage impact on its value compared to the other coordinates. It is also worth noting that the percentage impact of factors not included in the study was higher than the impact of most factors and interactions between them, amounting to Error = 15.96% for “L”; Error = 65.24% for “a”; Error = 17.25% for ‘b’. This was particularly evident in the case of the “a” coordinate.

## 3. Materials and Methods

### 3.1. Wood Impregnation

Sapwood samples of *Pinus sylvestris* L. and *Betula pendula* L. were used for the study. The dimensions of the samples were 75 mm × 25 mm × 0.4 mm, and their densities at 12% moisture content were 606 kg/m^3^ (pine) and 491 kg/m^3^ (birch), respectively. The wood was impregnated with an aqueous dispersion of graphene oxide (Advanced Graphene Products S.A., Zielona Gora, Poland) (concentration 0.08%). The graphene oxide suspension in water was subjected to ultrasonication (model PS-40A, CNC World Group Sp. z o.o., Jedlnia Letnisko, Poland) to reduce aggregating the graphene oxide flakes in solution. The samples were impregnated in a vacuum dryer model 1445-2 (Sheldon Manufacturing Inc., Cornelius, OR, USA) with a pump model V-700 (BUCHI Labortechnik AG, Flawil, Switzerland). Impregnation conditions were as follows: vacuum, 190 mbar; time, 30 min; temperature, 25 °C. After saturation, the wood samples were left in the solution for an additional 30 min. The retention of graphene oxide in the samples was determined by the differences between the initial weight of the samples and their weight after impregnation. Graphene oxide retention in pine and birch wood was 584 ± 32.16 kg/m^3^ and 560 ± 16.32 kg/m^3^, respectively. After impregnation, the wood samples were dried under controlled temperature and humidity conditions (temperature 20 ± 2 °C, relative humidity 30 ± 5%). The samples were maintained in these conditions for 4 weeks, with their weight monitored using a moisture analyzer (model MB 23, OHAUS Europe GmbH, Nänikon, Switzerland). Control samples, which were also impregnated, but only with water, were stored in the same way. The average moisture content of the samples before the UV aging test was as follows: pine, 6%; birch, 5.6%. After the aging process was completed, the samples were immediately subjected to analyses.

### 3.2. UV Aging

The UV aging process was carried out using a Solarbox 1500 chamber (CO.FO.ME.GRA, Milan, Italy) equipped with a 1500-watt xenon lamp. The irradiance was set to 550 W/m^2^, and the Black Standard Thermometer (B.S.T.) temperature was maintained at 80 °C. The exposure times were 16, 32, and 48 h, resulting in total radiation doses of 31, 62, and 93 MJ/m^2^, respectively.

### 3.3. FTIR Analysis

FTIR spectra were recorded using the total internal reflection (ATR) and diffuse reflection (Diffuse Reflection Spectroscopy) techniques in the range of 400/675–4000 cm^−1^, at a resolution of 4 cm^−1^ and a scan count of 64 on Thermo Scientific spectrometers (Nicolet IS50, ThermoFisher SCIENTIFIC, Waltham, MA, USA and Nicolet iN10 Microscope, Waltham, MA, USA). The spectra were baseline-corrected using Omnic 9.0 Software. FTIR tests were performed in 6 replicates.

### 3.4. Fluorimetric Analysis

**Figure 9:**
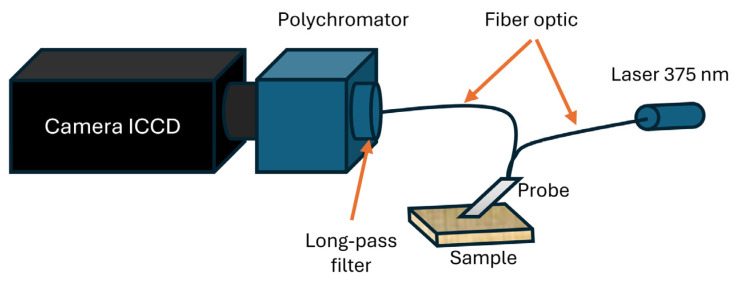
Laser-Induced Fluorescence setup.

Laser-Induced Fluorescence (LIF) spectra were measured using the laboratory setup shown in Figure 9. The tested veneer sample was placed on a flat surface. The measurement was performed by placing a fiber-optic probe (FCR-UV200/600-2-IND Avantes B.V., Apeldoorn, The Netherlands) on the surface. This probe allows the collection of fluorescence from the surface. Its head has an integrated system of two optical fibers used to deliver the excitation signal and to collect the emitted signal. The excitation source was a semiconductor laser from Power Technology Inc. (Alexander, AR, USA) generating CW (Continuous Wave) radiation with a wavelength of 375 nm and an output power of 16 mW. The excitation signal was directed using one of the fiber optic tips. The radiation emitted by the sample (fluorescence) was guided through the second fiber optic to a detection system consisting of an ICCD (Intensified Charge-Coupled Device) camera (Princeton Instruments PI-MAX 2, Trenton, NJ, USA) and coupled with a polychromator (Acton Research Corporation SpectraPro 2150i, Acton, MA, USA). A long-pass filter FF-407LP (Semrock Inc., Rochester, NY, USA) was placed in the emission optical path to remove the laser radiation. The integration time and signal gain were adjusted individually for each sample, depending on its fluorescent properties. A high signal-to-noise ratio was achieved by averaging 100 spectra. After the spectrum was recorded, a background measurement was performed with the same measurement parameters. To obtain a corrected spectrum, this signal was subtracted from the initially measured emission spectrum. To obtain the background signal, the tip of the probe was placed on the hole of the beam dump. The assessment of changes in fluorescence was performed for five replicates.

### 3.5. Evaluation of the Contact Angle

The contact angle of the wood samples was determined using a Haas Phoenix 300 goniometer (Surface Electro Optics, Suwon City, Republic of Korea). The volume of the water drop applied to the surface was 3 μL. An image analysis system (Image XP, Surface Electro Optics, version 5.8, Suwon City, Republic of Korea) was used to determine the angle between the tangent to the drop contour and the straight line intersecting its base. The contact angle value was calculated as the arithmetic mean of the angles measured on the left and right sides of the drop. Measurements were taken at intervals of 5, 20, 40, and 60 s from the moment the water drop was applied to the sample surface. The measurements were performed in 10 repetitions. The change in the contact angle relative to the starting point of the measurements was calculated according to the rule described in an earlier publication by the co-authors.

### 3.6. Color Measurement

The color change of control and impregnated wood samples exposed to UV radiation was determined using a Datacolor ColorReaderPRO spectrophotometer (Datacolor Technology, Suzhou, China). The data obtained were exported using the ColorReader application (Datacolor, Inc., Lawrenceville, NJ, USA). Ten measurements were taken for each sample surface. The following parameters were determined: L* (lightness), a* (chromaticity coordinate on the red-green axis), and b* (chromaticity coordinate on the yellow-blue axis). The measurements were performed in 20 repetitions. The total color difference (ΔE) was determined by ISO 7724-3:2003 [38]. The interpretation of the results was based on the following criteria:

0 < ΔE ≤ 1—imperceptible difference

1 < ΔE ≤ 2—difference noticeable to an experienced observer

2 < ΔE ≤ 3.5—difference noticeable to an inexperienced observer

3.5 < ΔE ≤ 5—noticeable difference

5 < ΔE—significant color change

### 3.7. Statistical Analysis

Statistical analyses were performed using STATISTICA 13.3 (TIBCO Software Inc., Palo Alto, CA, USA). Univariate general linear models (GLM) were used to assess the significance of the effects of experimental factors. The percentage contribution of each factor to the total variance was calculated based on the ratio of its sum of squares (SS) to the total SS in the ANOVA model.

## 4. Conclusions

This study investigated the impact of UV radiation on selected chemical and physical properties of wood surfaces. The following conclusions can be drawn:

1. Lignin is sensitive to photodegradation, as evidenced by changes in its absorption peaks at 1505 cm^−1^, 1592 cm^−1^, and 1456 cm^−1^.
2. Lignin in GO-coated samples also changed, indicating that UV radiation affects wood, even in the presence of GO.
3. When analyzing the effect of GO on the degree of oxidation of lignin irradiated with different doses of radiation, a slower increase in the C=O peak and a decrease in the C=C peak were observed in contrast to the control samples.
4. Exposing GO-impregnated wood to UV radiation reveals differences in surface wettability compared to control wood, which also undergoes aging. However, no significant effect of exposure time on differences in surface wettability was observed.
5. Exposing the wood surface to UV radiation intensifies the color change in impregnated wood.

The presented studies show that UV radiation is harmful to wood. Impregnation of wood with graphene oxide does not entirely protect against lignin photodegradation. However, there are some differences between the control wood and impregnated wood, which may suggest that graphene oxide partially reduces the rate of structural degradation of lignin, possibly due to its ability to absorb UV radiation or create protective barriers on the wood surface. The results emphasize the need for further research. The application of graphene oxide to the wood surface, which affects the effectiveness of wood protection against UV radiation, is of key importance in this context. Uniform application, layer thickness, and application technique may influence the interaction between graphene oxide and wood components.

## Figures and Tables

**Table 1:** The degree of change in the chemical composition of pine and birch as a change in the intensities of the C=C and C=O absorbance bands as a function of exposure time.

Exposure Time (h)	Lignin Degradation, Decrease in the C=C Band (R2)				Increase in the C=O Band (R1)			
	Without GO A_1505_/A_1370_		With GO A_1505_/A_1370_		Without GO A_1732_/A_1370_		With GO A_1732_/A_1370_	
	Birch	Pine	Birch	Pine	Birch	Pine	Birch	Pine
0	0.9506 ±0.0645	2.5357 ±0.8131	0.9008 ±0.05606	2.0192 ±0.1958	1.9430 ±0.05122	0.9199 ±0.2360	1.7552 ±0.0495	0.9736 ±0.4330
16	0.3901 ±0.06088	1.2643 ±0.7677	0.4151 ±0.05689	1.210 ±0.1912	2.5478 ±0.3822	2.3809 ±0.3.08	2.4469 ±0.1256	2.062 ±0.2131
32	0.1709 ±0.03664	0.9061 ±0.4115	0.2482 ±0.03690	0.833 ±0.000	3.0735 ±0.06459	3.3486 ±1.1312	2.9246 ±0.1212	2.553 ±0.3190
48	0.0696 ±0.00748	1.033 ±0.5415	0.1156 ±0.03691	0.7468 ±0.1250	3.2218 ±0.06481	2.939 ±0.3634	3.2706 ±0.2007	2.882 ±1.15

**Table 2:** Statistical analysis of the contact angle.

Factors	*p*	X (%)
Graphene impregnation (A)	1.55 × 10^−17^	1.52
Kind of wood (B)	<1.00 × 10^−17^	18.34
UV exposition time (C)	<1.00 × 10^−17^	6.36
Time after placing a droplet (D)	<1.00 × 10^−17^	44.38
A × B	7.60 × 10^−9^	0.74
A × C	<1.00 × 10^−17^	3.45
B × C	<1.00 × 10^−17^	3.10
A × D	1.87 × 10^−10^	1.09
B × D	2.22 × 10^−16^	1.77
C × D	1.15 × 10^−5^	0.87
A × B × C	<1.00 × 10^−17^	7.63
A × B × D	9.87 × 10^−1^	<0.01
A × C × D	8.10 × 10^−2^	0.33
B × C × D	7.24 × 10^−1^	0.13
A × B × C × D	5.30 × 10^−4^	0.65
Error		9.62

*p*—significant with α = 0.05; X—percentage of contribution.

**Table 3:** Statistical analysis of the coordinates of the color system.

Factors	L		a		b	
	*p*	X (%)	*p*	X (%)	*p*	X (%)
Graphene impregnation (A)	<1.00 × 10^−17^	72.46	<1.00 × 10^−17^	23.02	<1.00 × 10^−17^	40.24
Kind of wood (B)	<1.00 × 10^−17^	6.20	3.66 × 10^−5^	3.77	<1.00 × 10^−17^	11.01
UV exposition time (C)	1.34 × 10^−8^	2.21	1.48 × 10^−3^	3.39	<1.00 × 10^−17^	16.46
A × B	1.86 × 10^−1^	0.09	1.66 × 10^−3^	2.16	1.01 × 10^−7^	1.69
A × C	5.15 × 10^−2^	0.41	1.25 × 10^−1^	1.24	8.73 × 10^−3^	0.67
B × C	9.83 × 10^−4^	0.88	5.61 × 10^−1^	0.44	<1.00 × 10^−17^	12.19
A × B × C	4.62 × 10^−7^	1.78	3.33 × 10^−1^	0.73	3.47 × 10^−2^	0.50
Error		15.96		65.24		17.25

*p*—significant with α = 0.05; X—percentage of contribution.

## Data Availability

The original contributions presented in the study are included in the article; further inquiries can be directed to the corresponding author.
